# A pilot study on peritraumatic dissociation and coping styles as risk factors for posttraumatic stress, anxiety and depression in parents after their child's unexpected admission to a Pediatric Intensive Care Unit

**DOI:** 10.1186/1753-2000-3-33

**Published:** 2009-10-15

**Authors:** Madelon B Bronner, Anne-Marie Kayser, Hendrika Knoester, Albert P Bos, Bob F Last, Martha A Grootenhuis

**Affiliations:** 1 Psychosocial Department, Emma Children's Hospital Academic Medical Center, University of Amsterdam, The Netherlands; 2 Department of Paediatric Intensive Care, Emma Children's Hospital Academic Medical Center, University of Amsterdam, The Netherlands; 3 Department of Developmental Psychology, Vrije Universiteit, Amsterdam, The Netherlands

## Abstract

**Aim:**

To study the prevalence of posttraumatic stress disorder (PTSD), anxiety and depression in parents three months after pediatric intensive care treatment of their child and examine if peritraumatic dissocation and coping styles are related to these mental health problems.

**Methods:**

This is a prospective cohort study and included parents of children unexpectedly admitted to the Pediatric Intensive Care Unit (PICU) from January 2006 to March 2007. At three months follow-up parents completed PTSD (*n *= 115), anxiety and depression (*n *= 128) questionnaires. Immediately after discharge, parents completed peritraumatic dissocation and coping questionnaires. Linear regression models with generalized estimating equations examined risk factors for mental health problems.

**Results:**

Over 10% of the parents were likely to meet criteria for PTSD and almost one quarter for subclinical PTSD. Respectively 15% to 23% of the parents reported clinically significant levels of depression and anxiety. Peritraumatic dissocation was most strongly associated with PTSD, anxiety as well as depression. Avoidance coping was primarily associated with PTSD.

**Conclusion:**

A significant number of parents have mental health problems three months after unexpected PICU treatment of their child. Improving detection and raise awareness of mental health problems is important to minimize the negative effect of these problems on parents' well-being.

## Background

Stress reactions are common in parents in the aftermath of a life-threatening medical event of their child. However, a minority of parents develop chronic mental health problems [1]. Most common mental health problem after experiencing highly stressful events is posttraumatic stress disorder (PTSD), which is characterized by intrusive distressing memories, avoidance, emotional numbing and hyperarousal [2]. Other mental health problems may also be seen such as depression, anxiety disorder, sleep disturbances, and substance abuse [2]. Identification of parents with mental health problems after a child's life-threatening illness or injury is important. Once these parents are identified, psychological support can be offered at an early stage, aimed at minimizing chronic mental health problems and preserving their competence as caregivers. Consequently, parents will be able to support their child's recovery trajectory and adjustment in their best possible way. Therefore, improving identification and raise awareness of PTSD is a necessary first step in pediatrics.

Prevalence rates of mental health problems in parents vary widely after different life-threatening medical events. Research has mainly focused on cancer, diabetes and accidents with rates ranging from 10% to 40% for PTSD, anxiety and depression in parents [3-5]. Overall, women seem to have a higher risk of developing PTSD than men. Studies in heterogeneous pediatric intensive care treatment (PICU) populations have identified PTSD in approximately 13-27% of parents [6-10]. Prevalence rates of general psychological distress in PICU parents are even higher and exceed rates of distress of parents with children in general wards [7,10].

Risk factors for parental mental health problems are scarcely studied within PICU. Studies suggest that parental PTSD is not strongly related to objective characteristics of the PICU treatment but is related to parents' perceptions of the life threat for their child and to acute stress reactions in the PICU [6-10]. Research within pediatrics and traumatic stress studies has shown that responses immediately following the stressful event can help to predict the course of PTSD over time. For example, findings show that coping styles such as avoidance coping and passive reaction pattern have been linked to more mental health problems in pediatrics [11-13]. Furthermore, a recent meta-analysis of PTSD predictors in adults after interpersonal violence, combat and accidents suggested that peritraumatic psychological processes and peritraumatic dissocation are the strongest predictors of PTSD [14].

Peritraumatic dissocation is a state of limited or distorted awareness during and immediately after the stressful event. Examples of symptoms of peritraumatic dissocation are reduced awareness, time distortion, derealisation, amnesia or emotional numbing [15]. It has been suggested that such symptoms reflect a defensive response related to immobilization (freezing) in animals [16]. In addition, high levels of peritraumatic dissocation in adults during a stressful event such as interpersonal violence or burn injury, may also predict symptoms of psychopathology, such as anxiety and depression [17,18].

So far, only five studies examined prevalence of parental PTSD in heterogeneous PICU populations [6-10]. Anxiety and depression prevalence rates after PICU treatment have hardly been studied yet. Furthermore, until now no research has been conducted on whether coping styles or peritraumatic dissocation of parents after PICU treatment are risk factors of mental health problems such as PTSD, anxiety and depression. Therefore, the first aim of the present study was to describe the prevalence of mental health problems (PTSD, anxiety and depression) in parents three months after discharge from the PICU. The second aim of the study was to examine if coping styles and peritraumatic dissocation shortly after the stressful event are related to mental health problems in parents.

## Methods

### Patients

This is a prospective follow-up study three months after an unexpected PICU admission, focusing on physical and psychological consequences in children and their parents. In this study, we included *previously healthy *children, *unexpectedly *referred to the PICU for at least 24 hours with an acute life-threatening medical event. Children with known underlying illnesses or with scheduled elective surgery were excluded, as well as children admitted due to abuse or self-intoxication and the inability to complete Dutch questionnaires. The study was conducted from January 2006 to March 2007.

### Standardized transfer, aftercare program and procedure

This follow-up study is part of the standard aftercare program of the department of Pediatric Intensive Care. The objective of the aftercare program was to identify families (or family members) that need further physical or psychological support due to the unexpected PICU admission. The aftercare program comprised a standardized transfer out of the PICU to the pediatric general ward and a visit to the outpatient follow-up clinic at three months after discharge. In the standardized transfer by a PICU nurse, families were provided peritraumatic dissocation and coping questionnaires. Both parents were requested to complete the questionnaires and send them back to PICU. Written parental informed consent was obtained. The visit to the follow-up clinic consisted of a structured medical examination of the child by a pediatric physician, followed by a psychological screening by a psychologist. Prior to this clinic visit, parents received questionnaires on anxiety and depression as well as quality of life questionnaires concerning their children at home and were asked to bring them to this screening. During screening with the psychologist, parents completed PTSD questionnaires. The Medical Ethics Committee of the Academic Medical Centre in Amsterdam approved the study protocol.

### Outcome measures

PTSD in parents was measured with the Self-Rating Scale for PTSD (SRS-PTSD) [19]. The SRS-PTSD is a Dutch self-report questionnaire, and contains 17 items corresponding to *DSM-IV *diagnostic criteria for PTSD. The items are rated on a three-point scale: 0 = not at all; 1 = slightly/once/less than four times; 2 = very much/almost constantly/four times or more.

A symptom was rated as present if the item corresponding to the symptom scored 1 or higher, or in some cases 2 or higher. Total score of symptoms of PTSD was calculated on a continuous scale. This scale ranges from 0 (no symptoms at all) to 17 (all symptoms present). The diagnosis of PTSD is likely if at least one intrusive memory, three avoidance symptoms and two hyperarousal symptoms have been present in the previous four weeks. The diagnosis of subclinical PTSD is likely if at least one intrusive memory, one avoidance symptom and one hyperarousal symptoms were present in the previous four weeks. The SRS-PTSD demonstrated adequate psychometric properties. In general, the clinical utility and validity is satisfactory and the internal consistency is good. The instrument is regarded as a good alternative to the structured interview for PTSD, particularly at sites that have limited clinical resources [19,20]. In this study, the internal consistency (Cronbach's alpha) of the SRS-PTSD was .93.

Anxiety and depression in parents were measured with the Hospital Anxiety and Depression Scale (HADS) [21]. The HADS contains of a 7-item depression scale and a 7-item anxiety scale. The fourteen questions can be answered on a four-point scale (0-3), resulting in a range of 0-21 on each subscale. Higher total scores indicate more anxiety or depression in the past week. A cut-off score of 8 on both scales is considered as an indicator for clinically significant emotional distress for both men and women. The Dutch version of the HADS showed satisfactory validity and reliability on the total score and on the two subscales [22]. In this study, the internal consistency (Cronbach's alpha) of the anxiety scale was .87. The internal consistency (Cronbach's alpha) of the depression scale was .86.

### Risk factors

Generic coping in parents was measured with the Utrecht Coping List (UCL) [23]. This questionnaire measures general coping with stressful or problematic situations. The UCL covers seven coping styles: active problem focusing (AP), palliative reaction (PR), avoidance coping behaviour (AB), seeking social support (SS), passive reaction pattern (PP), expression of emotions (EE) and comforting cognitions (CC). The questionnaire contains 47 questions, which can be answered on a four-point scale, use of strategy 1 = rarely, 2 = sometimes 3 = often and 4 = very often. A higher score indicates more frequent use of the coping strategy at time of distressing experiences. The internal consistency and validity are satisfactory (20). In this study, the internal consistency (Cronbach's alpha) was AP: .75, PR: .74 AB: .65 SS: .87, PP: .68, EE: .59, CC: .64

Peritraumatic dissocation in parents was measured with the Peritraumatic Dissociative Experiences Questionnaire (PDEQ) [15]. PDEQ is a 10-item scale (range 10-50) that assesses the level of dissociative symptoms during or immediately after a stressful event (depersonalisation, derealisation, amnesia, altered body image and altered time perception). Items were rated on a scale ranging from 1 (not at all) to 5 (extremely true). The PDEQ has excellent psychometric properties; both validity and internal consistency are good [15]. In this study, the internal consistency (Cronbach's alpha) of the PDEQ was .86.

### Data analyses

The Statistical Package for Social Sciences (SPSS), Windows version 16.0, was used for all analyses. First, missing values were handled according to the guidelines given in the manuals of the questionnaires. Second, Mann-Whitney tests and Chi-square tests were completed to compare participants and non-participants with regard to child characteristics. In addition, parents that completed only outcome measures (SRS-PTSD and HADS) were compared with Mann-Whitney tests to parents that completed both outcome and risk measures (PDEQ and UCL-90). Third, prevalences of mental health problems (clinical and subclinical PTSD, anxiety, depression) in parents were calculated. Fourth, χ^2^-tests were used to examine differences in PTSD, anxiety, depression between mothers and fathers. Fifth, risk factors (peritraumatic dissocation and coping) for symptoms PTSD, anxiety and depression at three months after discharge from PICU were identified using univariate Poisson regression analyses. Then, a multivariate Poisson regression analysis was performed with entry significance level for risk factors of *p *< 0.20 in the univariate analysis. In addition, the multivariate model was corrected for gender. In both the univariate and multivariate analyses, generalized estimating equations (GEE) were used to correct for correlations in the response values of fathers and mothers from the same children [24]. An exchangeable working correlation matrix structure was assumed in the GEE procedure. For each regression, Wald Chi-Square values and their significance level were calculated to test the hypothesis whether the contribution (the regression coefficient (*B*)) of the entered variables significantly differed from zero.

## Results

### Participants

**Figure 1:**
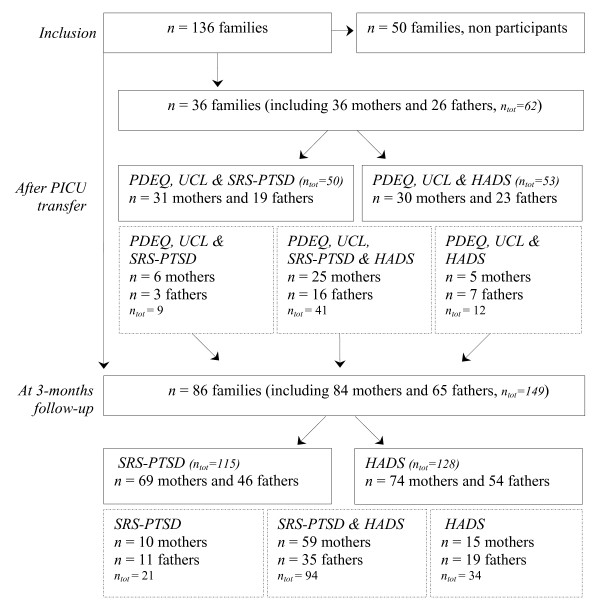
**Participating families (one or two parents living with a child) and number of completed questionnaires at follow-up and at PICU**. SRS-PTSD = Self-Rating Scale for PTSD; HADS = Hospital Anxiety and Depression Scale; PDEQ = Peritraumatic Dissociative Experiences Questionnaire; UCL = Utrecht Coping List.

In total, 136 families met the inclusion criteria for this study (Figure 1). Eventually, 86 out of 136 families visited the follow-up clinic at three months after PICU discharge and 36 families completed questionnaires immediate after PICU transfer. Fifty families did not participate in the study due to nonresponse, no show or refusal. The most common reasons given for not participating included the following: 'everything is going well', 'we have seen too many hospitals', 'we need some rest' and 'we don't want to remember that time'. No significant differences in child medical characteristics were found between families that participated in the study and that did not (Table 1).

**Table 1:** Child characteristics of the participating and non-participating families (n = 136)

	**Participants**	**Non-participants**	
	***n *= 86**	***n *= 50**	
	**Median (Range)**	**Median (Range)**	***p***
Age of child (years)	1.0 (0.0-17.0)	2.0 (0.0-16.1)	0.195
Length of stay in PICU (days)	4.5 (1.0-34.0)	4.0 (1.0-17.0)	0.254
Length of artificial ventilation (days)	2.0 (0.0-17.0)	1.0 (0.0-14.0)	0.172
Risk of mortality, PIM2 (%)	2.5 (0.2-58.9)	2.5 (0.2-28.7)	0.610

	***n*(%)**	***n *(%)**	

Gender of child			0.309
Female	27 (31.4)	20 (40.0)	
Male	59 (68.6)	30 (60.0)	
Artificial ventilation			0.203
No	19 (22.1)	16 (32.0)	
Yes	67 (77.9)	34 (68.0)	
Reason for PICU admission			0.515
Trauma	15 (17.4)	11 (22.0)	
Non-trauma	71 (82.6)	39 (78.0)	

PICU = Pediatric Intensive Care Unit

At three months follow-up, data of 149 parents (84 mothers and 65 fathers) were available (Figure 1). Of these 149 parents, 115 completed the PTSD questionnaire and 128 completed the anxiety and depression questionnaire. Thirty-four parents did not complete the PTSD questionnaire due to several reasons (e.g. parent did not visit follow-up clinic, questionnaire was not administered during screening). Twenty-one parents did not complete the anxiety and depression questionnaire mainly because parents forgot to bring the questionnaire to the follow-up clinic.

After the standardized transfer out of the PICU, 36 families returned peritraumatic dissociation and coping questionnaires (Figure 1). Data were available for 62 parents (36 mothers and 26 fathers). Final data for regression analyses included 50 cases of parents to examine risk factors for symptoms of PTSD, and 53 cases of parents for anxiety and depression. Moreover, parents that completed solely questionnaires at follow-up did not significantly score different on symptoms of PTSD (*U *= 1612.5, *n*_1_= 50, *n*_2 _= 65, *p *= 0.943), anxiety (*U *= 1874.5, *n*_1_= 53, *n*_2 _= 75, *p *= 0.583) and depression (*U *= 1871.5, *n*_1_= 53, *n*_2 _= 75, *p *= 0.569) than parents that completed questionnaires at both time measures.

### Mental health problems in parents at follow-up

In total, 12.2% of parents (*n *= 115) were likely to meet criteria for PTSD at three months follow-up, on top of that 24.3% were likely to meet criteria for subclinical PTSD (Table 2). Mothers had significantly more PTSD than fathers. Subclinical PTSD scores did not differ between mothers and fathers. Out of 128 parents, 23.4% reported possible clinically significant anxiety and reported 15.6% possible clinically significant depression (Table 2). Mothers scored significantly higher on the clinical score of anxiety than fathers. However, mothers and fathers did not significantly differ on the clinical score of depression. PTSD and anxiety (*r *= 0.75, p < 0.001) as well as PTSD and depression (*r *= 0.78, p < 0.001) correlated highly. Nineteen out of 86 families (22.1%; 11 mothers, 2 fathers, and 6 couples) that visited the outpatient follow-up clinic were referred for treatment or additional support after the psychological screening.

**Table 2:** Mental health problems in mothers and fathers three months after discharge from the PICU

	***n***	***n***	**Above cut-off (percent)**	**χ^2^**	***p***
PTSD	115	14	12.2%	4.392	0.036
Mothers	69	12	17.4%		
Fathers	46	2	4.3%		
Subclinical PTSD	115	28	24.3%	0.506	0.477
Mothers	69	15	21.7%		
Fathers	46	13	28.3%		
Anxiety	128	30	23.4%	3.884	0.049
Mothers	74	22	29.7%		
Fathers	54	8	14.8%		
Depression	128	20	15.6%	0.042	0.838
Mothers	74	11	14.9%		
Fathers	54	9	16.7%		

*Note*. Cut-off point subclinical PTSD: one intrusive memories, one avoidance symptom and one hyperarousal symptoms are reported. Cut-off point PTSD: one intrusive memories, three avoidance symptoms and two hyperarousal symptoms are reported. Cut-off point anxiety and depression: score of 8 and above for both mothers and fathers.

PTSD = posttraumatic stress disorder

### Peritraumatic dissocation, coping and mental health problems

In the univariate models, expression of emotions (B = 0.11, 95%CI -0.05 - 0.27, *p *= 0.168), avoidance coping (B = 0.07, 95%CI 0.03 - 0.10, *p *< 0.001), and peritraumatic dissocation (B = 0.05, 95%CI 0.03 - 0.08, *p *< 0.001) emerged as potential risk factors for symptoms of PTSD. Passive coping strategy (B = 0.06, 95%CI 0.01 - 0.11, *p *= 0.031), comforting thoughts (B = 0.07, 95%CI -0.00 - 0.13, *p *= 0.054), and peritraumatic dissocation (B = 0.04, 95%CI 0.02 - 0.06, *p *< 0.001) emerged as potential risk factors for anxiety. Expression of emotions (B = 0.15, 95%CI 0.01 - 0.29, *p *= 0.034), passive coping strategy (B = 0.10, 95%CI 0.05 - 0.15, *p *< 0.001) and peritraumatic dissocation (B = 0.04, 95%CI 0.02 - 0.07, *p *< 0.001) emerged as potential risk factors for depression.

Table 3 shows the final multivariate generalized estimating equations models with Poisson distribution of risk variables for symptoms of PTSD, anxiety and depression. Avoidance coping and peritraumatic dissociation were significantly related to symptoms of PTSD. Passive coping strategy, comforting thoughts and peritraumatic dissociation were significantly related to anxiety and peritraumatic dissociation was significantly related to depression.

**Table 3:** Multivariate Poisson regression coefficients for symptoms of PTSD, anxiety and depression predicted by coping and peritraumatic dissocation, corrected for gender

	**PTSD (*n *= 50)**			**Anxiety (*n *= 53)**			**Depression (*n *= 53)**		
			
	**B**	**95%CI**	***p***	**B**	**95%CI**	***p***	**B**	**95%CI**	***p***
Gender (female)	0.35	[0.04, 0.67]	0.027*	0.41	[0.14, 0.68]	0.003*	0.08	[-0.32, 0.48]	0.696
Active coping									
Expression of emotions	0.03	[-0.08, 0.14]	0.618				0.09	[-0.07, 0.25]	0.285
Palliative reaction									
Passive reaction pattern				0.06	[0.01, 0.11]	0.030*	0.06	[-0.00, 0.11]	0.064
Comforting thoughts				0.10	[0.01, 0.19]	0.029*			
Looking for social support									
Avoidance coping	0.05	[0.00, 0.11]	0.050*						
Peritraumatic dissocation	0.04	[0.01, 0.06]	0.001*	0.03	[0.01, 0.05]	0.007*	0.03	[0.00, 0.06]	0.045*

*p < 0.05 PTSD = posttraumatic stress disorder

## Discussion

This explorative study shows that 12.2% of parents were likely to meet diagnostic criteria for PTSD and on top of that 24.3% were likely to meet criteria for subclinical PTSD three months after PICU treatment. Respectively, 23.4% and 15.6% of parents reported possible clinically significant anxiety and depression. Mothers reported significantly more PTSD and anxiety than fathers did. Peritraumatic dissocation was related to mental health outcomes in general. Avoidance coping was primarily associated with PTSD. Furthermore, passive reaction pattern and comforting thoughts were significantly associated with anxiety.

The prevalence rate of parents that were likely to meet diagnostic criteria for clinical PTSD is similar to earlier research at PICU [6-10]. In addition, almost one quarter of parents were likely to meet criteria for subclinical PTSD which can lead to clinically meaningful levels of functional impairment as well [25]. Furthermore, in line with most studies on PTSD, the results show higher rates of PTSD in women than in men [26]. To our knowledge, the prevalence rates of anxiety and depression in parents of unexpectedly PICU admitted children have not been studied before. Compared to findings of a large national representative survey in The Netherlands the prevalence rates of mental health problems in the present study are considerably higher. In general, the 1-month prevalence of anxiety disorders in The Netherlands is 9.7%. Anxiety disorders are more prevalent than mood disorders. The 1-months prevalence of mood disorders is 3.9% in The Netherlands [27].

The avoidance coping strategy was strongly associated with symptoms of PTSD. PTSD symptoms increased as a function of using avoidance coping. This effect of avoidance coping has also been found in several earlier studies after cancer, and in general stress literature [11-13,26]. Interestingly, avoidance coping strategy was not related to anxiety and depression, indicating that avoidance coping may pose increased risk for specific posttraumatic stress reactions [11]. However, causality has not been established and avoidance coping may reflect a representation of the same underlying construct (e.g. overlap with avoidance symptoms of PTSD). Next to avoidance coping, peritraumatic dissocation also turned out to be significantly associated with symptoms of PTSD, as well as with symptoms of anxiety and depression. However, some recent studies suggest viewing the relationship between peritraumatic dissocation and PTSD as an artefact of confounding variables. In other words, peritraumatic dissocation is related to PTSD because it is associated with other risk factors such as prior mental health problems [28,29]. In sum, there seems to be a strong relationship between peritraumatic dissocation and mental health problems. Yet, this should not be interpreted as proof for a causal relationship and further prospective research is necessary to disentangle this connection [30].

The passive coping strategy was associated with anxiety and depression. The relationship between passive coping and mental health problems has been found in previous research after cancer, and in general stress literature as well [12,26]. Once again, this association may reflect a shared, underlying construct, or it may indicate a causal relationship with either distress affecting coping or coping affecting distress. If the association between passive coping and anxiety or depression is direct, this coping strategy could be seen as maladaptive. Passive coping may be related to the concepts of learned helplessness and locus of control [31]. These theories propose that perceived absence of control over the situation will lead to more negative mental health outcome. Helping parents manage the PICU period by regaining perceived control might be effective in reducing these outcomes (e.g. involve parents in the care for the child).

Some limitations of the study should be addressed. The first limitation of this study pertains to sampling issues. A considerable number of parents and children were lost due to non-response. Fifty families (37%) did not visit the outpatient follow-up clinic. This may have biased the results, even though similar response rates were found in earlier studies. The sample size for the regression analyses was even smaller. The timing of our study was not optimal since medical staff had to get used to the new standardized protocol of transfer out of the PICU. Our centre implemented this transfer protocol, of which the questionnaires were part of, in January 2006. Furthermore, we suspect that few parents were motivated to complete questionnaires immediately after PICU discharge due to possibly continuing stress of the hospital admission. Consequently, this small sample size raises questions about the generalizability of study findings and the degree to which study participants are representative of typical PICU populations. Therefore, findings of this study are preliminary and exploratory. Besides, it minimized the number of risk variables that could be included in the analyses of our study. Therefore, gender differences in peritraumatic dissocation and coping could not be analyzed. Second, a structured clinical interview can be regarded as the best measurement for mental disorders. The use of self-reports only gives an indication for the diagnosis of mental disorders and cut-off scores should be used with caution. Self-reports can lead to an overestimation of cases with mental health problems. Nevertheless, good diagnostic agreement between the SRS-PTSD self-report measure and clinical interviews for PTSD has been reported [19,20]. Third, in identifying risk factors for mental health problems of parents, other risk factors might be relevant, such as initial mental health problems, perceived life threat or previous stressful events. Future research should investigate multiple risk factors and their interactions in order to unravel the mechanisms underlying longer-term mental health problems.

Notwithstanding the limitations, the present study is one of the first longitudinal follow-up studies on anxiety, depression and PTSD in a relatively large number of mothers and fathers of children after unplanned PICU treatment, examining peritraumatic dissocation and coping. The results of this pilot study support that many parents experience symptoms of PTSD i.e. subclinical PTSD. Approximately one third of parents show clinical levels of PTSD, anxiety and depression after unexpected admission of their child at PICU for which adequate psychological support is necessary. Avoidance coping, passive reaction pattern and particularly peritraumatic dissocation were associated with mental health problems in parents. The presence of the variety of emotional reactions in the sample underscores the need for medical staff and psychosocial professionals to identify parents at risk and intervene in an early stage to minimize chronic and pathologic mental health problems. A next step is to replicate these findings in a larger sample of parents and explore possible other risk factors for mental health problems. So, parents at risk can be identified and monitored in an early phase and referred for psychological support if necessary.

Finally, this study can have some clinical implications for early identification of those parents at risk. The strong relationship between peritraumatic dissocation symptoms at PICU and parental mental health problems at three months suggests that pediatric health care providers in the hospital should ask parents about these peritraumatic dissocation symptoms. Inquiring parents during PICU admission about reduced awareness (do you ever lose track of what is going on around you?), time distortion (do you ever feel as though you are disoriented, as though you are uncertain about where you are of what time it is?), derealisation (do you ever feel as though you are a spectator, watching what is happening to you as if you were an outsider?), amnesia (can you remember everything of the PICU admision?) as well as emotional numbing (do you feel a restricted range of affect?) may help to identify those who are in need for further assessment and psychosocial support. This assessment is particularly warranted when the parent also applies passive and avoidance coping styles. In addition, a set of informational materials for use by pediatric health care providers has recently been developed: the medical traumatic stress toolkit [32]. This toolkit includes a preventative intervention model suggesting that the health care team provide every family with general information and basic support, and regularly screen for acute stress symptoms and risk factors to determine which children and families might need more support. This toolkit should be made accessible for parents and children at PICU and should be evaluated in future research for its effects on preventing or reducing PTSD, depression and anxiety .

## Competing interests

The authors declare that they have no competing interests.

## Authors' contributions

This study is part of an on-going explorative research program on physical and psychological consequences in children and their parents after an unexpected paediatric intensive care admission. MB had primary responsibility for the psychological screening of the families, data collection, data entry, all analyses and writing the manuscript. AK had a major contribution on data collection, data entry, and analyses. HK participated in the development of the program, had primary responsibility for the physical examination and contributed to the writing of the manuscript. This program is an initiative of two departments of the Emma Children's Hospital AMC, Amsterdam. APB is head of the paediatric intensive care unit and the fourth author, BFL is head of the psychosocial department. Both authors supervised the design and execution of the study, and contributed to the writing of the manuscript. Sixth author, MAG head research of the psychosocial department participated in the development of the program, supervised this study and final analyses, and contributed to the writing of the manuscript. All authors read and approved the final manuscript.
